# Added Value of Products from Endangered Local Sheep Breeds in Mountain Areas

**DOI:** 10.3390/ani14192855

**Published:** 2024-10-04

**Authors:** Elena Benedetti del Rio, Marco Berton, Nicolò Amalfitano, Maurizio Ramanzin, Enrico Sturaro

**Affiliations:** Department of Agronomy, Food, Natural Resources, Animals and Environment, University of Padova, Viale dell’Università, 16, 35020 Legnaro, Italy

**Keywords:** mountain farms, local sheep breeds, milk quality, meat quality

## Abstract

**Simple Summary:**

This study focuses on preserving four endangered local sheep breeds in the Italian eastern Alps, reared in small-scale farms. This study aims to assess the nutritional quality of their products (milk and meat) and explore ways to enhance their market value, thus supporting farmers economically. Results indicated that the milk and meat from the four breeds have unique qualities that could be promoted to increase consumer awareness and demand. By developing specific guidelines for the production and marketing of local sheep products, this research helps create a local supply chain based on the positive externalities that local breeds in mountain areas have on the environment.

**Abstract:**

Local sheep breeds in the Italian eastern Alps passed from ex situ to in situ conservation. These breeds are mainly reared by smallholders in low-input farming systems. To allow the sustainable use of genetic resources, the economic sustainability of farmers must be supported through production guidelines. Analyzing meat and milk composition and fatty acid profile, we aimed to characterize their products based on breed and diet to identify tailormade sales strategies. Results showed that both meat and milk have good nutritional values and can benefit from a pasture-based diet, irrespective of the breed. These results support the redaction of production guidelines based on the peculiar characteristics of these breeds: being multi-purpose breeds adapted to mountain areas and to grazing, thus contributing to the conservation of cultural and landscape heritage.

## 1. Introduction

The sustainable use of Animal Genetic Resources (AnGR) can increase the resilience of low-input farming systems to unpredicted changes (e.g., the spread of a disease or extreme weather conditions [1,2]), the adaptation to climate change [3], and support the provision of ecosystem services [4].

In the Italian Alps, sheep of local breeds were traditionally reared in mixed herds with cattle and goats in grassland-based farms [5]. However, the recent dramatic reduction in small, extensive farms [5] threatens the conservation of these breeds and their role as reservoirs of genetic diversity. In fact, the farms rearing them find it challenging to achieve their own sustainability due to different environmental, social, and economic constraints [6,7,8]. In this perspective, marketing strategies based on the intrinsic and extrinsic qualities of the product to raise consumer awareness can improve the added value of the products and support farmers in marginal areas [9].

The Alpagota, Brogna, Foza, and Lamon are local sheep breeds in the Italian eastern Alps, whose risk status is listed as “endangered” (Alpagota and Brogna) and “critical” (Lamon and Foza) by DAD-IS (Domestic Animal Diversity Information System) [10]. In the last years, the conservation of these breeds has evolved from ex situ in vivo to in situ in vivo, thanks to mating plans and genomic analysis, allowing local farmers to directly manage flocks in their farms or smallholders to keep them to support their conservation [11,12]. For each breed, an association of farmers has been formed, creating a collaborative community and developing its own main sales strategy: lamb for Alpagota (Slow Food Presidium), heavy lamb and sheep for both Brogna (Slow Food Presidium) and Foza, and lamb and sheep for Lamon. These breeds are reared in low-input farming systems, using local forage resources, such as hay or grazing on pasture.

To move from a simple conservation plan to a sustainable use of genetic resources, the farmers need support in implementing a local supply chain to guarantee their economic sustainability [13]. For this reason, it is fundamental to identify and characterize the relationships between intrinsic product quality and flock management to define production guidelines.

Therefore, our study aimed at highlighting potential breed differences in terms of nutritional indicators, such as proximate composition and fatty acid profile, offering a preliminary characterization of the products obtained from local breeds reared in situ in real farming conditions in the Italian eastern Alps. By surveying the quality of the products during the year, we could characterize the local supply chains and propose effective ways to add value to these products.

## 2. Materials and Methods

This survey was performed in north-eastern Italy from the winter period to the summer period of 2021 in the provinces of Belluno (46°8′ N, 12°12′ E), Verona (45°26′ N, 10°59′ E), and Vicenza (45°33′ N, 11°33′ E). We collected 21 bulk milk samples of Alpagota and Brogna and 45 meat samples (*longissimus lumborum*) of all breeds in different farms (Table 1). Sampling was constrained by the limited number of farms rearing these breeds [12] and reflected the actual production and local supply chain. Thus, the survey could not follow a balanced experimental design. The diet of the animals was completely forage-based or pasture-based. The collection began with hay-based samples (from February to March); pasture-based samples were collected from April to June. The chemical composition of meadow hay and pasture grass characterizing each breed area can be found in Table S1. Milk samples of Alpagota were available for hay-based and pasture-based diets. Brogna’s available samples only included pasture-based diets. Meat samples were available for the “lamb” (<4 months, males, live weight 16–35 kg), “heavy lamb” (4–12 months, males, live weight 25–45 kg), and “sheep” age classes (>12 months, females, live weight 45–85 kg), which were, however, not equally represented in all breeds because of the different sales strategies developed by each association of farmers. The analyzed records were collected after the animals were slaughtered in a commercial abattoir in compliance with European regulation No. 1099/2009 on the protection of animals at the time of killing [14]. The authors did not have direct control over the care of the animals included in this survey. Milk composition in terms of fat, protein, casein, lactose, total solids (TS), and not-fat solids (NFS) was determined through the MilkoScan FT2 Infrared Analyzer (Foss Electric A/S), while milk coagulation properties (MCP) in terms of rennet coagulation time (RCT), curd-firming time from RCT at 20 mm of curd firmness (k20), and curd firmness at 30 (a30), 45 (a45), and 60 (a60) minutes were obtained using the Formagraph (Foss Electric A/S, version 4.1) with the method described in Cipolat-Gotet et al. [15]. Milk samples were frozen and freeze-dried until fat was extracted. On the basis of each milk sample’s fat content, freeze-dried milk was weighed to obtain at least 40 micrograms of lipids.

Meat composition in terms of protein, fat, and ash was determined following AOAC [16]. The meat fat extraction process began after raw meat thawing at 4 °C for a night; then, fat was extracted with an accelerated solvent extractor (ASE, Thermo Fisher Scientific Inc., Waltham, MA, USA). After thawing, we weighed 4.0 g of meat and 15 g of Na_2_SO_4_ and mixed them with Hydromatrix (diatomaceous earth) into 10 mL stainless steel extraction cells for ASE 350 to obtain at least 40 micrograms of fat for a correct analysis through gas chromatography. The extraction solvent used was petroleum ether and the following extraction conditions: temperature of 125 °C; pressure of 1500 psi (10.3 MPa); static time 1 min; number of static cycles: 2; rinse; purge 60 s; total solvent 20 mL; total time 12 min (Dionex, Sunnyvale, CA, USA, Application Note 334). The solution obtained was heated at 45 °C for about 20 min under the N_2_ stream to allow all the solvent to evaporate. After 15 min in the oven at 60 °C, we let the vials cool under a vacuum and then weighed them to quantify the extracted fat, transformed into a percentage. Fatty acid methyl esters were obtained by a direct transesterification procedure, according to Christie [17], and successively analyzed using a 7820B GC System (Agilent Technologies, Santa Clara, CA, USA) fitted with an automatic sampler (Agilent 7693, Santa Clara, CA, USA) and a flame ionization detector connected to chromatography data system software (OpenLab CDS, Agilent ChemStation, version 2.4, Santa Clara, CA, USA). Separations were performed on SLB-IL 111 columns (200 m × 0.25 mm, 0.2 μm film thickness; Merck KGaA, Darmstadt, Germany), with a constant flow of hydrogen as carrier gas (1.6 mL/min). The temperature gradient was as follows: 80 °C for 1 min, then 50 °C/min to 170 °C, hold for 48 min; last phase for 6 °C/min up to 185 °C and maintained for 35 min. Fatty acid profiles were identified on 95% of the chromatogram. For statistical analysis, we categorized milk samples by combining breed and feeding (BxF) in three classes: “Alpagota hay” (AH), “Alpagota pasture” (AP), “Brogna pasture” (BP), and meat samples by combining breed and product type (BxPr) in five classes: “Alpagota lamb”, “Brogna heavy lamb”, “Foza heavy lamb”, “Lamon lamb”, and “sheep”. The relations between composition and quality variables of milk and meat were analyzed with principal components analysis (PCA; PROC PRINCOMP in SAS statistical software, version 9.4; SAS, 2013). Additionally, we tested the effects of BxF and BxPr as fixed effects on milk and meat variables with two mixed models (PROC MIXED; SAS, 2013), implementing the farm as a random effect. Milk and meat classes’ differences between least squares means were contrasted using a Bonferroni correction for multiple testing and considered significant with *p* < 0.05.

## 3. Results

### 3.1. Milk

Table 2 shows the descriptive statistics of meat and milk variables. Milk contained, on average, 7.8% fat, 6.2% protein (with nearly three-quarters composed of casein), and 4.3% lactose. The associated variability was moderate, with coefficients of variation (CV) ranging between 9% for lactose and 22% for fat. The fatty acid (FA) profile consisted of 68% saturated FA (SFA) and nearly 26% and 5% mono-unsaturated FA (MUFA) and polyunsaturated FA (PUFA), respectively. Omega-3 (n-3) and Omega-6 (n-6) were present at 1.3% and 2.4% (CV = 20.3 and 9.2), respectively, with an n-3/n-6 ratio of 1.90. Conjugated linoleic acid (CLA) was measured at 1.4% (CV = 37%). Regarding the milk coagulation properties (MCP), the rennet coagulation time (RCT) was achieved at 12 min on average and the curd firming time (k20) at 2.3 min after RCT; the curd firmness was recorded at 37, 31, and 26 mm at 30 (a30), 45 (a45), and 60 (a60) minutes of analysis, respectively, with a higher variability than that associated with milk composition traits (CV: 33–79%).

**Figure 1:**
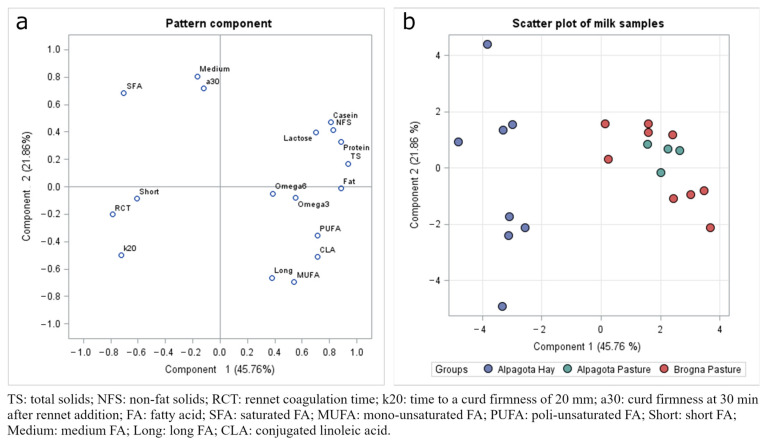
Principal component analysis of the milk collected from Alpagota sheep fed hay- and pasture-based diets and Brogna sheep fed pasture-based diets: (**a**) pattern component; (**b**) scatter plot.

The first component of the PCA explained nearly half of the variability, and the second component was slightly more than 21%. From the component pattern in Figure 1, we discussed those components positioned above 0.4 and below −0.4. All milk composition traits, unsaturated FA (MUFA and PUFA), CLA, and Omega3, were positively correlated with PC1; SFA, short FA, RCT, and k20 were negatively correlated. The SFA, medium FA, and a30 were positively correlated with PC2, whereas unsaturated FA, CLA, long FA, and k20 were negatively correlated. The scatter plot in Figure 1 shows that, according to PC1, the samples obtained at pasture (AP + BP) diverge from the samples obtained from in-house animals (AH).

ANOVA (Table 3) indicated that most quality traits were significantly affected by the interaction between breed and feeding system (BxFS). Table 3 shows the least squares means and results of the Bonferroni comparison only for the traits showing significant differences (*p* < 0.05); the complete results of the ANOVA are in Table S2. Milk from Alpagota and Brogna sheep at pasture had higher fat, protein, casein, lactose, total solids (TS), and non-fat solids (NFS) contents and better MCP than milk from Alpagota sheep on a hay-based diet. These results for Alpagota are in agreement with the better nutritional value of pasture, which showed lower neutral detergent fiber (NDF) and higher crude protein than meadow hay (Tables S1.1 and S1.2).

Regarding the FA profile, Brogna milk at pasture had a higher n-3 and a lower n-6/n-3 ratio compared to milk from both Alpagota sheep at pasture and those on a hay-based diet. Brogna milk at pasture had higher CLA contents than Alpagota milk on hay, with Alpagota milk at pasture being intermediate.

### 3.2. Meat

Table 2 provides the mean values and CV of meat quality traits. The meat had a dry matter content of about 24%, with 21% protein, 1.8% fat, and 1.1% ash. The lean components showed low variability (CV of 4% for protein and 5% for ash) compared to fat (CV = 91%). More than half of the FAs were SFAs, while MUFAs averaged 41% and PUFAs 6%. The n-3 and n-6 contents were 1.8% and 3.4%, with a n-6/n-3 ratio of 2.1. The variability in the FA profile was moderate (CVs from 6% to 37%), except for short FA content (CV = 44%), n-6/n-3 ratio (CV = 49%), and CLA content (CV = 76%).

**Figure 2:**
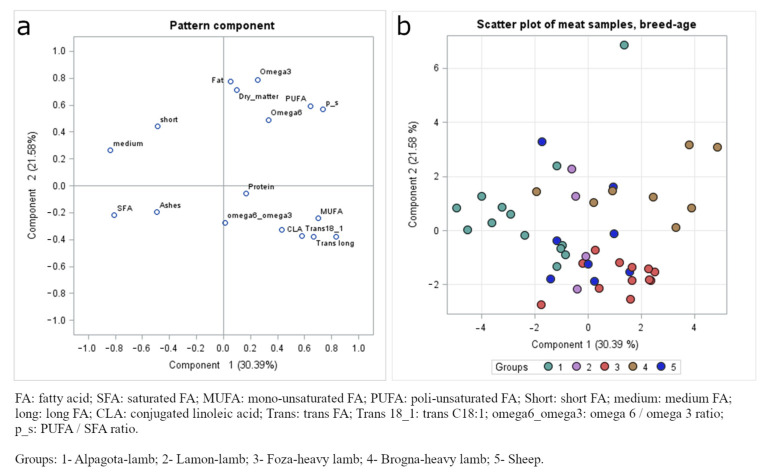
Principal component analysis of the meat collected from Italian eastern Alps local sheep breeds (Alpagota and Brogna): (**a**) pattern component; (**b**) scatter plot.

Figure 2 reports the PCA results for the meat quality traits. The first and second components explained nearly 31% and 22% of the total variability. The PUFA (and related ratio with SFA), MUFA, long FA, and Trans (plus Trans18:1) FA were positively correlated with PC1, whereas short and medium FAs, SFA, and ash content correlated negatively. Dry matter, fat, PUFA, n-3, and n-6 content, and PUFA:SFA ratio were positively correlated with PC2, and Long FA and Trans 18:1 correlated negatively. In contrast with what was observed in milk, there was no indication of clustering of quality traits according to breed and age.

When analyzed singularly in the ANOVA, the meat quality traits differed significantly between BxPr only for SFA, MUFA, short FA, n-6, and CLA (Table 4). Table 4 shows the least squares means and results of the Bonferroni comparison only for the traits showing significant (*p* < 0.05) differences; the complete results of the ANOVA are in Table S2. However, the variable showing a consistent and remarkable pattern of variation was CLA, which was much lower in lambs compared to older animals.

## 4. Discussion

### 4.1. Milk

Information on milk composition, yield, and coagulation properties is still very scarce for local sheep breeds in the Veneto Region. The results of a previous project regarding these four local breeds highlighted that, among them, milk production ranges from 0.8 to 1.2 L/day for around 3 months of lactation [18]. So, we can expect that multipurpose breeds would show higher fat and protein percentages than specialized breeds, such as the dairy Sarda breed, in agreement with Signorelli et al. [19]. In fact, the different levels of milk production due to different levels of specialization create a “dilution effect”, where milk production increases, but the absolute content of milk nutrients (as a percentage of milk dry matter) remains quite stable [20]. In our case, the flocks were kept indoors and fed hay at the beginning of lactation, when milk production increases, explaining the lower total solids content; on the contrary, the flocks on pasture were mostly in the middle or at the end of the lactation, explaining the higher total solids content. Of course, also the effect of shifting from hay to pasture is an important component that, in this in situ condition, cannot be disentangled from the lactation stage. However, in the context of the study, milk is mainly used to feed lambs. Just a few farms are organized to milk sheep, as outlined by our sample collection covering the whole supply chain of local breeds’ farmers. Indeed, to our knowledge, this study is the first to be conducted after the shift of the conservation management of these four Veneto sheep breeds at risk of extinction from ex situ to in situ.

In comparison with our results, Brogna, Foza, and Lamon milk examined by Bittante et al. [21] in ex situ experimental conditions had lower fat and protein contents and higher lactose contents, and the milk of the same breeds examined by Pellattiero et al. [22] had lower SFA and higher MUFA contents. These results suggest that the milk quality observed here, in real farm conditions, was rather good and suited for cheese-making.

The PCA and ANOVA found better composition and coagulation properties for pasture-based diets irrespective of the breed, which is a common result for milk [20,23]. In contrast, the FA profile seemed to vary more in dependence on the breed than feeding, whereas we expected the milk of sheep of both breeds on a pasture-based diet, and not only Brogna, to be richer in PUFA and CLA [24,25]. We may hypothesize that the duration of the period at pasture and/or the amount of grass ingested differed between the two breeds, with those of Alpagota being shorter and smaller, enough to exert short-term effects on milk fat and protein and MCP but not on FA profile. Other factors linked more to farming practices than to breeding, such as energy and protein diet concentration and animal productivity [25], might have influenced the differences observed. These breeds are kept by smallholders and part-time farmers [12] who do not follow shared technical guidelines and have different expertise.

### 4.2. Meat

In contrast with what was observed in milk, meat quality traits did not show clustering according to breed and product. When analyzed singularly, the meat quality traits differed significantly between BxPr only for a few variables: SFA, MUFA, short FA, n-6, and CLA (Table 3). Meat from ruminants represents one of the primary sources of CLA for humans, particularly when animals are reared on pasture [26,27]. CLA is biosynthesized in the rumen through bacterial isomerization and PUFA biohydrogenation [28]. Thus, the underdeveloped rumen in lambs may explain the lower production and deposit of CLA in the tissues concerning older animals, with more stable rumen activity.

The meat composition traits observed in this survey are in line with those found in Pellattiero et al. [29] in the ribeye samples of lambs from Brogna, Foza, and Lamon. What is remarkable in our results is the high variability of many traits, which was partially explained by the statistical analysis only for CLA. As suggested for milk quality, this variability is very likely linked to a lack of common farming and feeding practices.

### 4.3. Added Value of the Products

In order to add value to local production, it is fundamental to support the farmers for their commitment to preserve locally adapted breeds [30]. Because the products showed good intrinsic characteristics and, currently, no significant differences between breeds, it is necessary to support farmers through knowledge of the most suitable farming practices. Based on the results of this study, a pasture-based diet increases milk total solids. Therefore, cheese-making represents a possible way for farmers in mountain areas to diversify their offer and to valorize pasture as a local resource [31]. With regards to meat, its composition remains within the expected values in both hay- and pasture-based diets. Increased CLA content is shown when animals are grazing, supporting the added value of pasture for local sheep breeds in the Italian eastern Alps. The following step is to provide farmers with shared technical guidelines, focusing on the main characteristics of these breeds and raising consumers’ awareness [32,33]. In fact, as multi-purpose breeds adapted to mountain areas and grazing, their added value is the ability to contribute to landscape conservation, preventing grasslands from shrub encroachment and loss of biodiversity, and maintaining local cultural heritage [34]. This way, conservation objectives meet the sustainable use of genetic resources over time.

## 5. Conclusions

This study showed that the quality of the products is good and that there was no breed effect. Future studies might assess the quality per single product with the aim of achieving higher product standardization. Nevertheless, the added value provided by grazing on the quality of the products is particularly evident for milk. With regards to meat, the nutritional quality was in line with a previous study conducted ex situ, regardless of the diet.

These results on the intrinsic qualities of the products can be used in integration with the extrinsic quality (namely AnGR and landscape conservation) to develop marketing strategies aimed at raising consumer awareness using guidelines and product labels.

## Figures and Tables

**Table 1:** Number of farms involved and samples collected for milk (number of milk samples per breed and type of feeding) and meat (number of meat samples per breed and age class). NA: not available.

Breed	Farms	Milk Samples		Meat Samples		
		Hay-Based	Pasture-Based	Lamb	Heavy Lamb	Sheep
Alpagota	7	8	4	12	NA ^2^	2
Brogna	4	NA ^1^	9	NA ^2^	8	1
Foza	1	NA ^1^	NA ^1^	NA ^2^	12	NA ^2^
Lamon	5	NA ^1^	NA ^1^	5	NA ^2^	5

^1^ Not available due to the absence of farms milking under the specific conditions. ^2^ Not available due to the specific sale strategies of the farmers’ association.

**Table 2:** Descriptive statistics of composition and fatty acid profile of milk and meat, milk coagulation properties, and respective coefficients of variation (CV).

	Milk		Meat	
Variable	Mean	CV (%)	Mean	CV (%)
Composition (%)				
Fat	7.79	22.34	1.79	91.06
Protein	6.20	13.87	21.04	4.37
Casein	4.51	14.19	-	-
Lactose	4.29	9.32	-	-
Total solids	17.6	13.70	-	-
Non-fat solids	10.5	11.11	-	-
Ashes	-	-	1.11	5.41
Dry matter	-	-	24.33	6.99
Milk coagulation properties				
RCT (min)	11.8	78.60	-	-
k_20_ (min)	2.3	61.23	-	-
a_30_ (mm)	37.1	33.47	-	-
a_45_ (mm)	31.2	35.55	-	-
a_60_ (mm)	25.6	41.41	-	-
Fatty acids (FA; %)				
Saturated FA (SFA)	68.4	3.16	52.0	5.85
Mono-unsaturated FA (MUFA)	25.6	6.01	41.0	7.61
Poli-unsaturated FA (PUFA)	5.2	15.58	6.2	19.19
PUFA/SFA ratio	0.08	50.00	0.12	25.00
Short-chain FA	14.2	17.05	0.6	43.86
Medium-chain FA	45.5	6.93	34.9	13.92
Long-chain FA	56.7	7.30	63.7	8.78
Omega-3 (n-3)	1.28	20.31	1.8	29.55
Omega-6 (n-6)	2.38	9.24	3.4	24.05
n-6/n-3 ratio	1.90	7.40	2.1	48.57
Trans FA	5.81	11.58	5.9	32.54
Trans18:1 FA	39	10.15	4.2	37.08
Conjugated linoleic acid (CLA)	1.41	36.66	0.67	76.12

**Table 3:** Least squares means and results of the ANOVA on milk composition traits, milk coagulation properties, and fatty acid profile. Only variables differing significantly are shown.

Variable	Alpagota Hay	Alpagota Pasture	Brogna Pasture	*p*-Value	RMSE ^1^
Composition (%)					
Fat	6.01 ^c^	10.11 ^a^	8.35 ^b^	<0.001	0.77
Protein	5.23 ^b^	7.11 ^a^	6.65 ^a^	<0.001	0.35
Casein	3.83 ^b^	5.06 ^a^	4.87 ^a^	<0.001	0.35
Lactose	3.91 ^b^	4.49 ^a^	4.54 ^a^	<0.001	0.27
Total solids	14.85 ^c^	20.57 ^a^	18.71 ^b^	<0.001	0.71
Non-fat solids	9.26 ^b^	11.54 ^a^	11.20 ^a^	<0.001	0.6
Milk Coagulation Properties					
RCT (min)	21.39 ^a^	5.31 ^b^	6.21 ^b^	<0.001	5.49
k_20_ (min)	3.62 ^a^	1.32 ^b^	1.48 ^b^	<0.001	0.92
a_45_ (mm)	38.04 ^a^	17.95 ^b^	30.95 ^ab^	<0.01	8.76
Fatty acids (%)					
Saturated FA (SFA) ^2^	69.95 ^a^	68.00 ^ab^	67.22 ^b^	<0.05	1.84
Poli-unsaturated FA (PUFA) ^3^	4.62 ^b^	4.93 ^ab^	5.83 ^a^	<0.01	0.6
PUFA/SFA ratio	0.066 ^b^	0.073 ^ab^	0.087 ^a^	<0.01	0.01
Omega-3 (n-3)	1.15 ^b^	1.06 ^b^	1.50 ^a^	<0.001	0.18
n-6/n-3 ratio	2.03 ^a^	2.09 ^a^	1.70 ^b^	<0.001	0.14
Trans FA	4.06 ^b^	6.44 ^ab^	7.10 ^a^	<0.01	1.66
Conjugated linoleic acid (CLA)	1.04 ^b^	1.53 ^ab^	1.71 ^a^	<0.01	0.40

^1^ Root-mean-square error; ^2^ PUFA: from C16:2 to C22:6; ^3^ Short FA: from C6:0 to C10:1. ^a,b,c^ Least square means with different superscripts within each row differ significantly (*p* value < 0.05).

**Table 4:** Least squares means and results of the ANOVA on meat composition and fatty acid profile. Only variables differing significantly are shown.

Variable	Alpagota-Lamb	Lamon-Lamb	Foza-Heavy Lamb	Brogna-Heavy Lamb	Sheep	*p*-Value	RMSE
Fatty acids (%)							
Saturated FA (SFA) ^1^	53.82 ^a^	52.97 ^a^	51.94 ^ab^	49.15 ^b^	51.57 ^ab^	0.03	2.75
Mono-unsaturated FA (MUFA) ^2^	38.43 ^b^	40.27 ^ab^	42.19 ^a^	42.15 ^a^	42.58 ^a^	0.01	2.76
Short FA ^3^	0.83 ^a^	0.52 ^ab^	0.48 ^ab^	0.46 ^b^	0.48 ^b^	0.04	0.18
Omega-6 (n-6)	3.15 ^b^	4.87 ^a^	2.80 ^b^	4.15 ^ab^	3.26 ^b^	0.01	0.40
Conjugated linoleic acid (CLA)	0.29 ^c^	0.12 ^c^	1.25 ^a^	0.73 ^b^	0.55 ^c^	<0.001	0.32

^1^ SFA: from C6:0 to C20:0; ^2^ MUFA: from C10:1 to C22:1; ^3^ Short FA: from C6:0 to C10:1. ^a,b,c^ Least square means with different superscripts within each row differ significantly (*p* value < 0.05).

## Data Availability

The data presented in this study are available upon request from the corresponding author upon reasonable request.

## Supplementary Materials

The following supporting information can be downloaded at: https://www.mdpi.com/article/10.3390/ani14192855/s1, Table S1.1: Chemical composition (% of dry matter, DM) of meadow hay used in the different breed areas. Table S1.2: Chemical composition (% of dry matter, DM) of grass from pasture grazed in the different breed areas. Table S2: Results of the ANOVA on composition and fatty acid profile of milk and meat, and milk coagulation properties.
