# Supplementary material for: Added Value of Products from Endangered Local Sheep Breeds in Mountain Areas
Source: Animals (Basel). 2024 Oct 4;14(19):2855. doi: 10.3390/ani14192855 (PMC11475488; doi:10.3390/ani14192855)
Supplement: Supplementary file 1 [file animals-14-02855-s001.zip › Table S1.pdf]

Table S1.1 Chemical composition (% of dry matter, DM) of meadow hay used in the different breed areas.

	Alpagota		Brogna		Foza		Lamon	
Variable	Mean	SD	Mean	SD	Mean	SD	Mean	SD
Crude Protein (% DM)	12.53	0.35	14.26	3.33	15.57	9.82	13.14	5.48
Fat (% DM)	2.22	0.32	3.51	0.45	3.2	0.21	3.33	0.65
Neutral detergent fiber (% DM)	51.36	3.63	52.13	10.3	51.18	9.4	46.68	14.81
Ash (% DM)	8.97	3.62	9.57	1.79	7.57	2.28	11.13	3.72

Table S1.2 Chemical composition (% of dry matter, DM) of grass from pasture grazed in the different breed areas.

	Alpagota		Brogna		Foza		Lamon	
Variable	Mean	SD	Mean	SD	Mean	SD	Mean	SD
Crude Protein (% DM)	18.18	3.9	19.93	6.04	15.94	4.09	16.51	5.14
Fat (% DM)	2.99	0.14	3.51	0.53	3.16	0.49	3.44	0.5
Neutral detergent fiber (% DM)	42.96	2.02	51.86	5.6	54.24	1.84	48.71	4.9
Ash (% DM)	11.47	1.71	9.02	1.57	8.23	2	8.8	1.11
