# Supplementary material for: Added Value of Products from Endangered Local Sheep Breeds in Mountain Areas
Source: Animals (Basel). 2024 Oct 4;14(19):2855. doi: 10.3390/ani14192855 (PMC11475488; doi:10.3390/ani14192855)
Supplement: Supplementary file 1 [file animals-14-02855-s001.zip › Table S2.pdf]

Table S2. Results of the ANOVA on composition and fatty acids profile of milk and meat, and milk coagulation properties.

	Milk		Meat	
Variable	F-value	P-value	F-value	P-value
Composition (%)				
Fat	42.22	<0.001	0.61	0.66
Protein	53.08	<0.001	1.47	0.24
Casein	25.36	<0.001	-	-
Lactose	12.63	<0.001	-	-
Total solids	105.42	<0.001	-	-
Non-fat solids	29.64	<0.001	-	-
Ashes	-	-	2.33	0.08
Dry matter	-	-	0.95	0.45
Milk coagulation properties				
RCT (min)	19.69	<0.001	-	-
k_{20} (min)	14.12	<0.001	-	-
a_{30} (mm)	0.38	0.69	-	-
a_{45} (mm)	7.02	0.006	-	-
a_{60} (mm)	3.47	0.054	-	-
Fatty acids (FA; %)				
Saturated FA (SFA)	4.78	0.02	3.09	0.03
Mono-unsaturated FA (MUFA)	1.94	0.17	4.45	0.006
Poli-unsaturated FA (PUFA)	9.24	0.002	2.49	0.06
PUFA/SFA ratio	8.99	0.002	2.03	0.11
Short-chain FA	3.5	0.052	2.84	0.04
Medium-chain FA	0.17	0.85	1.12	0.36
Long-chain FA	0.75	0.49	1.22	0.32
Omega-3 (n-3)	12.22	<0.001	2.47	0.07
Omega-6 (n-6)	3.63	0.05	4.61	0.005
n-6/n-3 ratio	16.04	<0.001	0.19	0.94
Trans FA	7.45	0.004	2.54	0.06
Trans18:1 FA	0.30	0.74	0.89	0.48
Conjugated linoleic acid (CLA)	6.26	0.008	10.28	<0.001
